# Comparing the effect of clopidogrel versus ticagrelor on coronary microvascular dysfunction in acute coronary syndrome patients (TIME trial): study protocol for a randomized controlled trial

**DOI:** 10.1186/1745-6215-15-151

**Published:** 2014-05-01

**Authors:** Sang-Don Park, Yong-Soo Baek, Seong-Ill Woo, Soo-Han Kim, Sung-Hee Shin, Dae-Hyeok Kim, Jun Kwan, Keum-Soo Park

**Affiliations:** 1 Department of Internal Medicine, Inha University Hospital, 7-206, 3-GA Sinheung-Dong, Jung-gu, Incheon 400-711, South Korea

**Keywords:** MeSH terms, Myocardial infarction, Antiplatelet agents, Microcirculation

## Abstract

**Background:**

Although prompt reperfusion treatment restores normal epicardial flow, microvascular dysfunction may persist in some patients with acute coronary syndrome (ACS). Impaired myocardial perfusion is caused by intraluminal platelets, fibrin thrombi and neutrophil plugging; antiplatelet agents play a significant role in terms of protecting against thrombus microembolization. A novel antiplatelet agent, ticagrelor, is a non-thienopyridine, direct P2Y12 blocker that has shown greater, more rapid and more consistent platelet inhibition than clopidogrel. However, the effects of ticagrelor on the prevention of microvascular dysfunction are uncertain. The present study is a comparison between clopidogrel and ticagrelor use for preventing microvascular dysfunction in patients with ST elevation or non-ST elevation myocardial infarction (STEMI or NSTEMI, respectively).

**Methods/design:**

The TIME trial is a single-center, randomized, open-label, parallel-arm study designed to demonstrate the superiority of ticagrelor over clopidogrel. A total of 152 patients with a spectrum of STEMI or NSTEMI will undergo prospective random assignment to clopidogrel or ticagrelor (1:1 ratio). The primary endpoint is an index of microcirculatory resistance (IMR) measured after percutaneous coronary intervention (PCI); the secondary endpoint is wall motion score index assessed at 3 months by using echocardiography.

**Discussion:**

The TIME trial is the first study designed to compare the protective effect of clopidogrel and ticagrelor on coronary microvascular dysfunction in patients with STEMI and NSTEMI.

**Trial registration:**

ClinicalTrials.gov: NCT02026219. Registration date: 24 December 2013.

## Background

The novel antiplatelet agents, prasugrel and ticagrelor, have overcome many of the pharmacodynamic limitations of clopidogrel and have improved outcomes in patients with acute coronary syndrome (ACS) [1,2].

Ticagrelor is a non-thienopyridine, direct P2Y12 blocker that is more potent than clopidogrel and is associated with less inter-individual variability. In the PLATO trial, it was found to be superior to clopidogrel with respect to cardiovascular outcomes and total mortality without increasing the risk of bleeding [2].

Faster onset of action and more potent and reversible receptor bindings are possible explanations for the superior outcomes of ticagrelor [3]. In addition to the potent inhibition of platelet function, ticagrelor has previously been shown to increase adenosine levels by inhibiting adenosine re-uptake at the tissue level and inducing adenosine triphosphate (ATP) release from human red blood cells, which stimulate vasodilation [4]. A recent study confirmed that ticagrelor increased adenosine plasma concentration in patients with ACS when compared with clopidogrel [5].

Since the microvascular dysfunction is multifactorially developed, involving intraluminal platelets, fibrin thrombi, neutrophil plugging, vasoconstriction, myocyte contracture, local interstitial edema and intramural hemorrhage [6-8], antiplatelet agents have been used as a basic medication for protecting against microvascular dysfunction [9,10].

However, there has been no data on the role of higher plasma adenosine and stronger inhibition of platelets in the protection against microvascular dysfunction in ST elevation or non-ST elevation myocardial infarction (STEMI or NSTEMI, respectively). Thus, we have designed a clinical study that will compare the protective effect of ticagrelor and clopidogrel on microvascular dysfunction in patients with STEMI and NSTEMI. In our study, microvascular dysfunction will be assessed by using the index of microcirculatory resistance (IMR), which correlates closely with microvascular damage in myocardial infarction [11].

## Methods

### Study design

The TIME trial is a single-center, randomized, open-label, parallel-arm study designed to demonstrate the superiority of ticagrelor compared with clopidogrel treatment in preventing microvascular dysfunction in patients with STEMI and NSTEMI.

**Figure 1:**
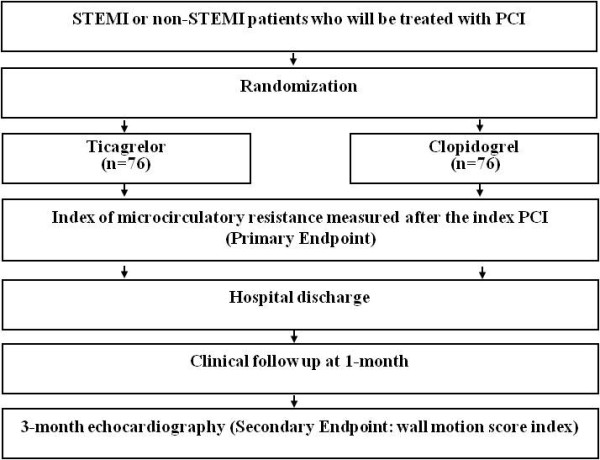
**TIME trial algorithm.** Non-STEMI, non-ST-segment elevation myocardial infarction; PCI, percutaneous coronary intervention; STEMI, ST-segment elevation myocardial infarction.

The protocol of the trial has been registered at ClinicalTrials.gov (NCT02026219), and a brief flowchart of the entire study is summarized in Figure 1. The schedule of events for this trial is described in Table 1.

**Table 1:** Schedule of events

**Variable**	**Baseline**	**Post-procedure**	**Follow-up**	
			**30 days**	**3 months**
			**± 2 weeks**	**± 1 month**
Medical/clinical history (age, sex, risk factors, clinical diagnosis, angina status, cardiac history)	X			
Informed consent^a^	X			
Inclusion/exclusion criteria	X			
Brief physical examination	X			
Vital status	X		X	X
Weight, height	X			
12-lead ECG^b^	X	X		X
Angiogram^c^	X			
IMR examination		X		
CBC	X			X
Electrolytes, LFT	X			X
Creatinine, BUN	X			X
hs-CRP	X			X
Fasting plasma triglycerides, HDL cholesterol, total cholesterol	X			X
Fasting glucose level^d^	X			X
HbA1c^e^	X			X
Medications	X		X	X
CK, CK-MB, troponin I^f^	X	X		
proBNP	X			X
Echocardiography	X			X

^a^The informed consent will be signed either prior to or after the diagnostic angiogram; ^b^additional ECGs will be performed at 60 ± 30 minutes post-procedure. ECG at follow-up visits will only be obtained when clinically indicated by symptoms such as recurrent chest pain, ischemia or significant arrhythmias, heart failure or other signs or symptoms of clinical instability; ^c^routine follow-up angiography will be recommended at 9 months, but it can be performed at 9 months ± 2 months. Unscheduled angiograms ≥6 months after index procedure will be considered as the 9-month follow-up angiogram in final analysis; ^d^might be measured later, before discharge, when the patient is in a fasting state; ^e^will be performed in patients with diagnosed diabetes mellitus; ^f^cardiac enzyme levels should be followed up for at least 24 hours in patients with symptoms such as recurrent chest pain, ischemia, significant arrhythmias, heart failure or other clinical signs or symptoms of cardiac instability. Otherwise, the decision is up to the operator. If follow-up is performed, enzyme levels must be measured every 8 hours for at least 24 hours post-index procedure. BUN, blood urea nitrogen; CBC, complete blood count; CK, creatine kinase; ECG, electrocardiogram; HbA1c, hemoglobin A1c; HDL, high-density lipoprotein; hs-CRP, high sensitivity C-reactive protein; IMR, index of microcirculatory resistance; LFT, liver function test; pro-BNP, pro-brain natriuretic peptide.

### Endpoints

The primary endpoint of the study is an IMR measured immediately after index percutaneous coronary intervention (PCI). The secondary endpoint is the echocardiographic finding of left ventricular wall motion score index at 3 months after the index PCI.

### Patient population

We will enroll 152 patients, comprising 76 patients in the ticagrelor group and 76 patients in the clopidogrel group. Patients of at least 18 years of age, who have STEMI or NSTEMI with documented ischemia due to a significant lesion in a native coronary artery, will be included in this study. All inclusion and exclusion criteria are summarized in Appendix A and Appendix B, respectively.

If all the inclusion criteria are met and none of the exclusion criteria apply, the patients will be asked to provide written informed consent, as required by the institutional review board and in accordance with the Declaration of Helsinki.

### Randomization and interventions

After the patients are enrolled, drug randomization will be performed by using a random number table that will be independently managed at the Inha University Hospital Cardiovascular Research Center (Incheon, South Korea).

### Percutaneous coronary intervention (PCI)

After random assignment to clopidogrel or ticagrelor treatment, the index PCI procedure must be carried out on all patients within 48 hours. All patients will receive 300 mg aspirin and a loading dose of 600 mg clopidogrel or 180 mg ticagrelor before the procedure, unless the patient has been taking one of these medications for at least 1 week prior to the procedure. Unfractionated heparin will be administered intravenously in boluses to maintain an activated clotting time of >250 seconds during the procedure. Administration of glycoprotein IIb/IIIa inhibitors will be based on the physician’s discretion. PCI will be performed according to current international guidelines. The goals of the procedure are to achieve optimal angiographic efficacy of PCI at the infarct-related artery and minimize the risk of procedure-related complications. A full range of commercially available guiding catheters, balloon catheters and guide wires will be readily available.

### Index of microcirculatory resistance (IMR) measurements

IMR measured at the infarct-related artery after primary PCI is a strong predictor of myocardial damage [11] and will provide a quantitative assessment of microvascular dysfunction in ACS patients [12]. We plan to assess the microvascular dysfunction using IMR measured at the infarct-related artery immediately after reperfusion therapy.

The IMR will be assessed immediately after index PCI by using methods described previously [13,14]. An intracoronary combined pressure-temperature sensor-tipped guide wire (Radi PressureWire 5; Radi Medical Systems, Uppsala, Sweden) will be used to measure the thermodilution-derived IMR and fractional flow reserve (FFR). The pressure sensor will be placed at the distal two-thirds of the infarct-related artery. Intracoronary nitroglycerine will be administered (200 μg). Hyperemia will be induced by using an adenosine infusion (140 μg/kg⋅min) administered via the femoral or antecubital vein. Aortic and distal coronary pressures will be measured during hyperemia, and the FFR will be calculated by using the formula: FFR = Distal coronary pressure/Aortic pressure. The IMR will be calculated from the ratio of the mean distal coronary pressure at maximal hyperemia to the inverse of the hyperemic mean transit time (*T*_mn_) as follows: IMR = Distal pressure × *T*_mn_ during hyperemia [13,15]. Vessels with severe stenosis (distal coronary pressure ≤60 mmHg) and collateral flow will be excluded from the analysis because of the effect of collateral flow [16,17].

### Post-PCI medication

All patients included in this trial will be treated according to the current American College of Cardiology (ACC)/American Heart Association (AHA) guidelines regarding post-stenting management, which specify treatment with at least 100 mg of aspirin daily, and 75 mg clopidogrel or 90 mg ticagrelor daily for at least 12 months after PCI.

### Follow-up

Clinical follow-up will be performed at specified time points (Table 1). Follow-ups should be office visits, but telephone contact will be allowed. Data collected during all follow-up visits will include angina class and the presence of major adverse ischemic, neurologic or bleeding events, including re-hospitalization, re-catheterization and adverse events/serious adverse events (SAEs). Original source documents must be submitted for any clinical events (death, reinfarction, revascularization, stroke or any other SAE within the 9-month follow-up). If the patient is readmitted to a non-study hospital, all possible efforts should be made to obtain original source documents from that hospital. For all reinfarctions, ECGs and cardiac enzymes (creatine kinase (CK), CK-MB and troponin) must be obtained and recorded.

### Sample size calculation

The objective is to determine whether ticagrelor is superior to clopidogrel in preventing microvascular dysfunction in patients with STEMI or NSTEMI. Based on previous reports for IMR values in STEMI and NSTEMI [11,18,19], we will assume that the mean IMR in the clopidogrel group is approximately 30 U with a standard deviation of 20 U. To demonstrate that ticagrelor is superior to clopidogrel, we will assume a reduction in IMR of more than 10 U. Taking into account a drop-out rate of 20% of the randomized patients, we will need 152 patients, comprising 76 in the ticagrelor group and 76 in the clopidogrel group, with 80% power to detect a group difference of 10 U in the change of the IMR value at a two-sided alpha-level of 0.05.

### Statistical analyses

All primary and secondary endpoints will be analyzed both on an intention-to-treat basis (all patients analyzed as part of their assigned treatment group) and on a per protocol basis (patients analyzed as part of their assigned treatment group only if they actually received their assigned treatment).

Multivariate predictors of all primary and secondary endpoints will be determined by using multivariate regression models. Forward stepwise selection algorithms will be used to select independent predictors. Baseline characteristics of study patients will be summarized in terms of frequencies and percentages for categorical variables and by using means with standard deviations for continuous variables. Categorical variables will be compared by using Fisher’s exact test. Continuous variables will be compared by using the two-sample *t*-test. A *P* value of 0.05 will be established as the level of statistical significance for all tests. Major subgroup analyses of the primary and major secondary endpoints will be performed, including patients with diabetes mellitus, advanced age (age ≥70 years) and the diagnosis (STEMI or NSTEMI).

### Trial organization

#### Executive committee

The executive committee will comprise the study chairperson and the principal investigators of the investigating center. This committee will approve the final trial design and protocol that will be issued to the Data and Safety Monitoring Board (DSMB) and the clinical site. This committee will also be responsible for reviewing the final results, determining the methods of presentation and publication, and the selection of secondary projects and publications by members of the steering committee.

#### Data Safety Monitoring Board (DSMB)

The DSMB is composed of general and interventional cardiologists. The DSMB will function in accordance with applicable regulatory guidelines. The board members are independent and will not be participating in the trial. The DSMB committee will review the safety data from this study, and make recommendations based on safety analyses of unanticipated device effects, SAEs, protocol deviation, device failures and 30-day follow-up reports. The frequency of the DSMB meetings will be determined prior to study commencement. Additionally, the DSMB may call a meeting at any time if there is reason to suspect that safety is an issue.

All cumulative safety data will be reported to the DSMB and reviewed on an ongoing basis throughout enrollment and follow-up periods to ensure patient safety. Every effort will be made to allow the DSMB to conduct an unbiased review of patient safety information. All DSMB reports will be made available to the appropriate agencies upon request but will otherwise remain strictly confidential.

#### Clinical events adjudication committee (CEAC)

The clinical events adjudication committee (CEAC) is comprised of interventional and non-interventional cardiologists who are not participants in the study. The CEAC is charged with the development of specific criteria to be used for the categorization of clinical events and clinical endpoints in the study, which are based on protocol. At the outset of the trial, the CEAC will establish explicit rules outlining the minimum amount of dates required and the algorithm to be followed in order to classify a clinical event. All members of the CEAC will be blinded to the primary results of the trial.

The CEAC will meet regularly to review and adjudicate all clinical events in which the required minimum data is available. The Committee will also review and rule on all deaths that occur throughout the trial.

### Ethical approval

This study has been approved by the institutional review board of Inha University Hospital.

## Discussion

Although prompt reperfusion treatment restores normal epicardial flow, microvascular dysfunction may persist in some patients with ACS [20,21]. The presence of microvascular dysfunction is considered a poor prognostic factor in patients with ACS [22-24].

Impaired myocardial perfusion is caused by intraluminal platelets, fibrin thrombi, neutrophil plugging, vasoconstriction, myocyte contracture, local interstitial edema and intramural hemorrhage [6-8]. There have been a number of studies in patients with ACS in which the use of antiplatelet agents and vasodilators attenuated myocardial damage [9,10,25-28]. Such findings strongly support the contribution of thrombotic and vasoconstrictor mechanisms leading to the development of impaired microcirculation. Antiplatelet agents play a relevant role in protecting against thrombus microembolization.

Recently the novel antiplatelet agents, ticagrelor and prasugrel, have exhibited greater, more rapid and more consistent platelet inhibition than clopidogrel. Ticagrelor is a non-thienopyridine, direct P2Y12 blocker that is more potent than clopidogrel and is associated with less inter-individual variability. In the PLATO trial, ticagrelor was found to be superior to clopidogrel with respect to cardiovascular outcomes and total mortality without increasing the risk of bleeding [2].

The dual antiplatelet regimen including aspirin and clopidogrel has been used as the standard antiplatelet modality in patients with ACS; however, clopidogrel has several negative features. For example, the phenomenon of the variability of response to clopidogrel, causing elevated platelet reactivity, is seen in approximately 15 to 30% of patients, and it has been linked to ischemic events including stent thrombosis after PCI. Another issue is the delayed onset of action [29-31] that may be observed with clopidogrel treatment. These drawbacks have led to the development of more potent and rapid-acting antiplatelet agents. Compared with clopidogrel, ticagrelor causes greater, faster and more consistent platelet inhibition [2].

Beside the potent effect of inhibition of platelet function, ticagrelor has been demonstrated previously to increase adenosine levels by inhibiting adenosine re-uptake at the tissue level and inducing ATP release from human red blood cells, which stimulate vasodilation [4]. In addition, a recent *in vivo* study in patients with ACS showed a significant increase in adenosine plasma concentration in patients treated with ticagrelor. In previous studies, adenosine has shown multiple mechanisms affecting platelet function, vessel tone and microcirculation. Adenosine inhibits platelet aggregation through the activation of adenosine 2A receptors and also dilates coronary microvessels through the activation of adenosine 2A receptors, which are strongly implicated in the regulation of coronary blood flow [32,33]. However, the role of adenosine in protecting the ischemic heart remains controversial and the long-term impact of high plasma adenosine concentration on coronary artery disease remains to be determined [34]. These properties of adenosine may be particularly important in the context of diseased vessels and the ischemia associated with ACS.

Consequently, based on the potent antiplatelet effect and pleiotropic properties mediated by the increased adenosine, we postulate that ticagrelor will reduce the microvascular dysfunction in patients with ACS compared with clopidogrel. Therefore, we designed a clinical study that will evaluate the protective effect on microvascular dysfunction of ticagrelor compared with clopidogrel in patients with STEMI and NSTEMI.

## Trial status

Enrollment into the TIME trial started in September 2013. The study is expected to be completed by the end of August 2014.

## Appendix A

Inclusion criteria:

• Patients with STEMI or NSTEMI with documented ischemia due to a significant lesion in a native coronary artery

• Patients eligible for coronary revascularization through PCI

• Patients must be ≥18 years of age

• Patients who are mentally and linguistically able to understand the aim of the study and show sufficient compliance in following the study protocol

• Patients are able to verbally acknowledge an understanding of the associated risks, benefits and treatment alternatives to therapeutic options of this trial. The patients, by providing informed consent, agree to these risks and benefits as stated in the patient informed consent document.

## Appendix B

Exclusion criteria:

• Unprotected left main artery

• Culprit lesion at side branch

• Stent thrombosis

• High-degree atrioventricular block

• Cardiogenic shock

• Cardiac arrest

• Contraindication to adenosine

• Pregnancy

• History of cerebrovascular accident or myocardial infarction within 1 year.

## Abbreviations

ACC: American College of Cardiology; ACS: Acute coronary syndrome; AHA: American Heart Association; ATP: Adenosine triphosphate; CEAC: Clinical events adjudication committee; CK: Creatine kinase; DSMB: Data and Safety Monitoring Board; FFR: Fractional flow reserve; IMR: Index of microcirculatory resistance; NSTEMI: ST-segment non-elevation myocardial infarction; PCI: Percutaneous coronary intervention; SAE: Serious adverse event; STEMI: ST-segment elevation myocardial infarction.

## Competing interests

The authors declare that they have no competing interests.

## Authors’ contributions

SD and YS conceived the work, undertook data collection and analysis, and wrote the manuscript. DH and SHS designed the work and critically revised the manuscript. SI and SHK undertook data collection and analysis. JK and KS designed the work, undertook data collection, and critically revised the manuscript. All authors read and approved the final manuscript.
